# Reciprocal Relationships Among Household Chaos, Parenting Stress, and Children's Behavioral Self‐Regulation From Early to Middle Childhood

**DOI:** 10.1111/famp.70053

**Published:** 2025-06-17

**Authors:** Qingyang Liu, Ying Zhang, Rachel A. Razza

**Affiliations:** ^1^ Department of Human Development and Family Science Syracuse University Syracuse New York USA; ^2^ Department of Psychology Clarkson University Potsdam New York USA

**Keywords:** behavioral self‐regulation, cross‐lagged panel model, household chaos, longitudinal design, parenting stress, reciprocal associations

## Abstract

Household chaos has been shown to be negatively associated with children's behavioral functioning and relational processes. Behavioral self‐regulation, the ability to manage emotions, behaviors, and attention in response to contextual demands, could be particularly vulnerable to chaotic home environments. Parenting stress, the negative psychological responses to caregiving challenges, could also be exacerbated in chaotic environments. However, the complex interactions among these factors, specifically how household chaos, parenting stress, and children's development of behavioral self‐regulation mutually influence one another, remain underexplored. Grounded in the transactional framework, this study used longitudinal data from the Future of Families and Child Wellbeing Study (*N* = 4195) to examine the reciprocal relationships among these constructs during the transition from early to middle childhood (ages 3, 5, and 9). Results suggested reciprocal associations between household chaos and children's behavioral self‐regulation from age 3 to 5 and a similar bidirectional link between parenting stress and children's behavioral self‐regulation during the same developmental stage. In middle childhood, household chaos at age 5 predicted behavioral self‐regulation at age 9. These findings highlight the need for interventions to mitigate household chaos and alleviate parenting stress to foster children's long‐term behavioral self‐regulation development.

## 1 Introduction

Human development and behaviors are widely understood to be the result of dynamic interactions between individuals and their environments (Bronfenbrenner and Morris 2007). One key aspect of the family environment is household chaos, characterized by high levels of disorganization, noise, crowding, and unpredictable daily routines (Wachs and Evans 2010). Household chaos can overwhelm children with random stimuli and disruptions (Andrews, Atkinson, et al. 2021; Andrews, Dunn, et al. 2021) and has been linked to adverse cognitive, emotional, and behavioral outcomes (Marsh et al. 2020; Martin et al. 2012; Vernon‐Feagans et al. 2016). Among the key developmental capacities that could be undermined by household chaos is children's behavioral self‐regulation, which supports children's school readiness and lifelong success (Blair and Raver 2015; Robson et al. 2020). Children exposed to household chaos exhibited lower levels of self‐regulation (Crespo et al. 2019; Liu, Razza, et al. 2024; Liu, Zhang, et al. 2024). Furthermore, household chaos is associated with elevated parenting stress (Bodrij et al. 2021; Spinelli et al. 2021), which may further compromise the development of adaptive self‐regulation during childhood (Harmeyer et al. 2016; Planalp et al. 2022).

At present, the complex interactions among these factors, specifically how household chaos, parenting stress, and children's behavioral self‐regulation mutually influence one another, remain underexplored. A transactional framework highlights the necessity of examining these developmental processes as bidirectional, shaped by ongoing exchanges between individuals and their contexts. From this perspective, children are active agents in shaping their contexts (Sameroff 2009). Children's difficulties in behavioral self‐regulation may exacerbate the household chaos and elevate parenting stress, while disorganized environments and high parenting stress may further hinder behavioral self‐regulatory skills, creating a cycle of dysregulation. These dynamics may also operate between household chaos and parenting stress, as unpredictable home environments heighten parental stress, and parental stress in turn intensifies instability. Importantly, the experience and interpretation of household chaos (Dumas et al. 2005; Evans et al. 2005) and parenting stress (Cassells and Evans 2017; Nomaguchi and House 2013; Yan 2022) are shaped by families' racial, ethnic, and socioeconomic contexts. To account for the contextual diversity, this study utilizes longitudinal data from the Future of Families and Child Wellbeing Study, a racially and socioeconomically diverse sample by design, to examine reciprocal relationships among household chaos, parenting stress, and children's behavioral self‐regulation from early to middle childhood. Understanding these intricate and possibly bidirectional interactions has substantial implications for developmentally informed preventive interventions to support children's self‐regulation and promote family flourishing.

### 1.1 Family Context and the Development of Self‐Regulation

Behavioral self‐regulation is a multidimensional construct that reflects children's ability to monitor, inhibit, and modulate physiological arousal, attention, and emotional responses, enabling observable behaviors in response to environmental demands (Nigg 2017). Extensive research has demonstrated that behavioral self‐regulation serves as a key protective factor for school readiness, academic achievement, and long‐term health and psychosocial functioning (Blair and Raver 2015; Guedes et al. 2023; Hennessy et al. 2020; Perry et al. 2018; Robson et al. 2020). As children grow, behavioral self‐regulation tends to increase from early childhood to adulthood and stabilize during later life stages (Liu, Razza, et al. 2024; Liu, Zhang, et al. 2024; Montroy et al. 2016; Park and Dearing 2025), making its early development essential for future success (Robson et al. 2020). Early childhood, a critical period for the development of high‐order cognitive functioning (Blair 2016; Diamond 2013; Iruka 2025; Thompson and Steinbeis 2020), enables children to master, hone, and internalize behavioral self‐regulation with the help of external co‐regulation from caregivers (Wesarg‐Menzel et al. 2023). As children transition into formal school settings and middle childhood, they are expected to increasingly regulate their behaviors independently, shifting from externally guided compliance to internally motivated self‐regulatory processes (Grant 2025; Hong et al. 2021; Sameroff 2010).

Behavioral self‐regulation is highly malleable and deeply influenced by family dynamics (Girod et al. 2025; Li et al. 2024; McClelland et al. 2015). The developmental systems framework emphasizes that children's capacity to self‐regulate behavior is shaped by the interrelated ecological family systems in which they are embedded (Lerner et al. 2006). The home environment is the most proximal context during early childhood (Bronfenbrenner and Morris 2007), playing a critical role in burgeoning self‐regulatory capacity (Bagais and Pati 2023; Ku and Blair 2021; Oloye and Flouri 2021). Through dynamic interactions with caregivers in the home context, children learn to regulate their behavior based on modeling, feedback, and responses they receive (Gee 2020; Kopp 1982; Paley and Hajal 2022). Building on the developmental systems framework metatheory, the present study adopts the transactional framework (Sameroff 2009) to guide the analysis of how behavioral self‐regulation both shapes and is shaped by the family environment over time. This analytic lens emphasizes the bidirectional, recursive processes through which children and caregivers mutually influence one another across developmental periods.

### 1.2 Household Chaos and Behavioral Self‐Regulation

Bronfenbrenner's Process–Person–Context–Time (PPCT) Model emphasizes the dynamic interplay between household chaos and developmental outcomes (Bronfenbrenner and Morris 2007). Within this model, the home environment is considered as the microsystem that plays a pivotal role in shaping both parents and children through daily interactions. Within the home environment, chaos can be viewed as a context with disordered activity manifested by noise, crowding, disorganization, and unpredictability (Wachs and Evans 2010). The chaotic home environments can disrupt parent–child interactions and negatively impact children's developmental outcomes (Marsh et al. 2020). Household chaos poses a significant threat to the development of behavioral self‐regulation across childhood. As such, chaotic home environments with overstimulation, instability, and unpredictable disruptions can overwhelm children in higher‐order cognitive processing, making it challenging for them to establish consistent self‐regulatory capacities (Andrews, Atkinson, et al. 2021; Andrews, Dunn, et al. 2021). The lack of established family routines further hampers the development of behavioral self‐regulation (Selman and Dilworth‐Bart 2023), as routines provide a critical structure for practicing self‐regulation (Bridley and Jordan 2012). Children in chaotic environments may receive inconsistent demands, which could diminish their capacity to develop effective self‐regulatory strategies.

While the PPCT framework helps illuminate how household chaos undermines self‐regulatory abilities (Andrews, Atkinson, et al. 2021; Andrews, Dunn, et al. 2021; Martin et al. 2012; Vernon‐Feagans et al. 2016), it is less often used to explain how children may influence and shape their environments over time. Alternatively, the transactional framework offers an important extension by emphasizing the bidirectional nature of developmental processes (Sameroff 2009). It is plausible that children with behavioral self‐regulation difficulties may struggle to follow routines, transition smoothly, or manage frustration, potentially contributing to greater household disorder. Understanding the development of this bidirectional relationship is crucial for designing interventions that target child self‐regulation and contextual factors, offering opportunities to break this cycle at critical developmental stages. Notably, it is important to consider the broader socioeconomic and cultural context in understanding both the perception and impacts of household chaos. Household chaos could be shaped by structural factors, such as poverty, with greater economic hardship linked to higher levels of household chaos (Brown et al. 2019; Garrett‐Peters et al. 2016; Liu, Razza, et al. 2024; Liu, Zhang, et al. 2024). Additionally, racial and ethnic variations exist in how household chaos is perceived and experienced. These differences may be related to cultural norms supporting multigenerational living arrangements, urban crowding, and cultural preferences for sounds or activity levels (Cherry and Gerstein 2023; Wachs and Corapci 2003).

### 1.3 Parenting Stress and Behavioral Self‐Regulation

Bronfenbrenner's PPCT framework emphasizes that relational processes, such as parenting stress, can influence the development of children's well‐being (Bronfenbrenner and Morris 2007). Parenting stress describes negative psychological responses to the challenges of raising and caring for children (Deater‐Deckard 1998). Elevated parenting stress can increase the risk of behavioral self‐regulation development throughout childhood (Blair 2010; Planalp et al. 2022). Parents living in chaotic environments may experience heightened stress as they struggle to establish new routines, often expecting their children to regulate their behaviors more effectively amid a disordered context. Higher levels of parenting stress have been linked to greater behavioral problems, aggression, and self‐regulation deficits in children both in low‐income minoritized families and in upper‐middle‐class White families (Cook et al. 2024; Gee et al. 2018; Suh and Luthar 2020). Conversely, lower parenting stress, combined with greater home stability, enables parents to engage in more warmth, responsive, and sensitive parenting practices (Marsh et al. 2020), essential for fostering children's behavioral self‐regulation (Kopp 1982). For example, Lee et al. (2023) used the data from the Future of Families and Child Wellbeing Study (FFCWS), a sample that included more low‐income families, revealing that parenting stress at age five was a significant predictor of children's internalizing and externalizing behaviors at the same age. Similarly, Choi and Becher (2019) used the same dataset and found a significant association between parenting stress at age five and subsequent behavioral problems in children at age nine, highlighting the potential long‐term impact of early parenting stress.

While parenting stress has been identified as a trigger for maladaptive behavioral self‐regulation in children, emerging evidence suggests that children's self‐regulatory abilities can also shape parenting processes. For example, using data from the FFCWS, Liu et al. (2023) found that children with better self‐regulation elicited more parental warmth and less harsh parenting during the transition from early to middle childhood. Similarly, Jiang et al. (2023) employed cross‐lagged panel models to examine reciprocal associations between parenting stress and child behavior problems between ages three and five, revealing that earlier child behaviors predicted later parenting stress. However, no studies using the FFCWS dataset to date have examined whether these reciprocal dynamics extend specifically to behavioral self‐regulation. Notably, these dynamic processes occur within larger socioecological contexts. Social locations, families' identities, and socioeconomic status could have shaped the opportunity for stabilization in a chaotic home. For instance, families from cultural backgrounds such as Asian, Black, and Latine experiencing poverty may experience more parenting stress due to intersecting contextual stressors (Cassells and Evans 2017; Nomaguchi and House 2013; Wu et al. 2025; Yan 2022). Moreover, child sex could influence parental stress levels, such that parents of boys often report higher parenting stress compared to parents of girls (Fang et al. 2024). Additionally, the adult‐to‐child ratio is associated with parenting stress and the resources each child receives. Higher ratios typically correlate with reduced stress and better resource allocation per child (Hong and Liu 2021; Jiang and Fung 2022).

### 1.4 Household Chaos and Parenting Stress

Household chaos can disrupt parent–child interactions and trigger parenting stress (Marsh et al. 2020). Specifically, household chaos can undermine parents' ability to provide consistent and responsive caregiving (Cherry and Gerstein 2023; Coldwell et al. 2006), heightening psychological stress levels as they adapt to unpredictable environments (Dumas et al. 2005). While extensive literature has examined how household chaos impacts parenting behaviors, such as increased hostile parenting (Tucker et al. 2018), reduced warmth (Valiente et al. 2007; Vernon‐Feagans et al. 2016), and diminished responsiveness (Andrews, Atkinson, et al. 2021; Andrews, Dunn, et al. 2021; Cherry and Gerstein 2023), less attention has been given to its direct influences on parenting stress. Chaotic environments may intensify stress as parents are forced to repeatedly adjust plans or routines, creating a sense of overwhelm. Over time, household chaos could become more persistent, leading to long‐term psychological stress (Bodrij et al. 2021). Prolonged exposure to a chaotic environment increases the risk of elevated parenting stress, particularly as children mature through critical developmental stages that require consistent parenting, which becomes more complicated in disordered home settings. Such efforts in empirical studies that examined preschoolers' behavioral regulation through the lenses of poverty and parenting are evident in the work of Liu, Razza, et al. (2024); Liu, Zhang, et al. (2024). Interestingly, their findings indicated that household chaos at age three did not correlate with maternal parenting stress at the same age or behavioral regulation at age five, suggesting that such effects might be indicated at a different age rather than solely during early childhood.

Evans and Wachs (2010) proposed that household chaos and parenting processes should be viewed as mutually reinforcing, reflecting evolved family dynamics. However, most studies have focused on how household chaos triggers parenting stress (Coldwell et al. 2006) while overlooking the possibility that high levels of parenting stress might also contribute to more chaotic environments. Parents experiencing significant parenting stress may struggle to maintain consistent routines, inadvertently creating disorganized or unpredictable environments that exacerbate their stress. This reciprocal relationship is consistent with a transactional framework (Sameroff 2009), which posits that contextual factors like household chaos and relational processes like parenting stress are dynamically interlinked. Recognizing the underlying bidirectional associations between chaotic home environments and parenting stress could inform intervention programs that foster healthy developmental home contexts for children's growth. In addition to family‐level processes, it is important to recognize the broader systemic barriers minoritized parents face, such as limited access to affordable childcare, unpaid parental leave, job insecurity, and financial hardship (Garland McKinney and Meinersmann 2023). While not directly examined in this study, these exosystem‐level constraints may cascade into the home environment, exacerbating parenting stress and household chaos. Moreover, contextual factors, such as socioeconomic status, culture, and race/ethnicity, should also be considered when interpreting the dynamics between household chaos and parenting stress, as perceptions and experiences of these processes may differ across racial and cultural groups (Cassells and Evans 2017; Wachs and Corapci 2003).

### 1.5 Current Study

This study aims to elucidate the associations among contextual factors (i.e., household chaos), relational processes (i.e., parenting stress), and child developmental outcomes (i.e., behavioral self‐regulation). Specifically, the present study examines the reciprocal relationships among these constructs from early childhood to middle childhood. To the best of our knowledge, this is the first study to simultaneously assess these family dynamics within the same models, providing a comprehensive understanding of how household chaos and parenting stress interact with behavioral self‐regulation across time. Distinct from prior FFCWS studies that have examined reciprocal associations between parenting stress and child behavior problems, the current study is the first to test bidirectional associations specifically involving children's behavioral self‐regulation across developmental transitions, while simultaneously accounting for household chaos as a broader contextual factor. We have developed the following research aims and hypotheses: (1) Based on the developmental systems and transactional frameworks, we hypothesize that household chaos and children's behavioral self‐regulation will have reciprocal influences across ages 3, 5, and 9. (2) Similarly, we hypothesize that parenting stress and children's behavioral self‐regulation will also exhibit bidirectional effects across ages 3, 5, and 9. (3) Grounded in Evans and Wachs (2010) proposed theory, we hypothesize that household chaos and parenting stress will mutually reinforce each other across ages 3, 5, and 9.

## 2 Method

### 2.1 Participants and Procedure

The current study utilized data from the Future of Families and Child Wellbeing Study (FFCWS). The participant recruitment and assessment procedures were approved by the Institutional Review Boards at Columbia University and Princeton University. Informed consent, both verbal and written, was obtained from participants during each wave of data collection. The dataset used in the present study is publicly accessible through the Office of Population Research at Princeton University. As this study involved secondary data analysis, it qualified for exemption from additional institutional ethical board review. The FFCWS is a national longitudinal study that tracks a birth cohort born across 20 U.S. cities with a population of 200,000 or more. By design, the FFCWS oversampled the children born to unmarried mothers (*N* = 3711), who were at greater risk of family instability and living in poverty, compared to 1187 children born to married parents (Reichman et al. 2001). The first wave of data (baseline) was collected at the time of the children's birth between 1998 and 2000, with subsequent waves collected when children were ages 1, 3, 5, 9, 15, and 22 years old. In this study, we utilized data from three collection periods: when the children were aged 3 (collected between 2001 and 2003), aged 5 (collected between 2003 and 2006), and aged 9 (collected between 2007 and 2010).

The original sample consisted of 4898 mother–child dyads. The current study was restricted to families who had at least one valid data point in household chaos, parenting stress, or behavioral self‐regulation, leaving a sample size of 4195. As shown in Table 1, 52.36% of children (*N* = 2006) were male. At year 3, the average age of mothers was 28.22 (*SD* = 6.06). On average, each household included two adults and two children. The majority of families were predominantly socioeconomically disadvantaged and racially diverse, with mostly Black (48.07%) or Latine (26.19%). Just over half of the mothers reported educational levels of high school or below (56.32%) and living in poverty below 200% of the federal poverty line (66.92%).

**TABLE 1:** Descriptive Statistics for Demographic (*N* = 4195).

Variable	Sample Mean (SD) or Count (%)
Child sex	Boys = 2006 (52.36%)
Child's age at age 3 (months)	35.76 (SD = 2.57)
Child's age at age 5 (months)	61.73 (SD = 2.78)
Child's age at age 9 (months)	112.51 (SD = 4.47)
Mother's age at age 3 (years)	28.22 (SD = 6.06)
Poverty Categories (age 3)	
0%–49% of the poverty line	951 (22.58%)
50%–99% of the poverty line	813 (19.31%)
100%–199% of the poverty line	1054 (25.03%)
200%–299% of the poverty line	571 (13.56%)
> 300+ of the poverty line	822 (19.52%)
Mother Race	
White	921 (21.93%)
Black	2019 (48.07%)
Latine	1100 (26.19%)
Others	164 (3.81%)
Mother's Education (age 3)	
Less than high school	1170 (27.80%)
High school or equivalent	1200 (28.52%)
Some college, technical	1307 (31.06%)
College or graduate school	531 (12.62%)
Marital Status (age 3)	
Single	2775 (65.96%)
Married	1432 (34.04%)
Adult to Child Ratio	2:2

### 2.2 Measures

#### 2.2.1 Household Chaos

Household chaos was measured by five items across years 3, 5, and 9 from the Home Observation for Measurement of the Environment (HOME; Bradley and Caldwell 1984). During in‐home observation, the observer rated the chaotic levels of the home environment. The five items assessed by the interviewer were whether the household was rated as (1) crowded, (2) cluttered, (3) unclean, (4) unsafe for young children, and (5) noisy. Following prior research, ratings on these five items were used as indicators to create the latent factors for household chaos at each time point (Kamp Dush et al. 2013).

#### 2.2.2 Parenting Stress

Parenting stress was measured by the four items across years 3, 5, and 9 drawn from the Parent Stress Inventory (Abidin 1990). Mothers self‐reported the amount of parenting stress brought on by changes in employment, income, or other factors in the parents' life. The parenting stress measure has been widely used in prior FFCWS research (Chiang and Bai 2024; Cook et al. 2024; Cooper et al. 2009). The items included “I often feel tired, worn out, or exhausted from raising a family,” “Being a parent is harder than I thought it would be,” “I feel trapped by my responsibilities as a parent,” and “I find that taking care of my child(ren) is much work than pleasure.” Ratings on these four items were used as indicators to create the latent factors for parenting stress at each time point.

#### 2.2.3 Behavioral Self‐Regulation

Children's behavioral self‐regulation was measured by maternal report using the Child Behavioral Checklist (CBCL) 2–3 for age 3, CBCL 4–18 for age 5, and CBCL 6–18 for age 9 (Achenbach 2011). In the current study, the same 14 items were selected at each time point to reflect children's behavioral self‐regulatory skills, which have established validity in previous research (Jackson et al. 2018; Liu et al. 2023; Watters and Wojciak 2020; Zhang et al. 2024). Mothers were asked to rate the frequency of children's behavior on a three‐point Likert scale, in which *not true* (0), *somewhat or sometimes true* (1), and *very true or often true* (2). The current study reverse coded all items such that higher scores indicated better behavioral self‐regulation. An example item included “He/She can't concentrate, can't pay attention long.” Ratings on these fourteen items were used as indicators to create the latent factors for behavioral self‐regulation at each time point.

#### 2.2.4 Control Variables

Several demographic variables correlated with household chaos, parenting stress, and children's behavioral self‐regulation were included in the model. Family poverty status was the mother's self‐reported household income when children were at age 3, further divided by the federal poverty threshold of that year, adjusted for family size. Five categories were included as 0%–49% of the federal poverty threshold (1), 50%–99% of the federal poverty threshold (2), 100%–199% of the federal poverty threshold (3), 200%–299% of the federal poverty threshold (4), and above 300 + % of the federal poverty threshold (5). The current study dummy coded these categories by dividing the groups into those *below 200% of the federal poverty threshold group* (0) and those *at or* *above 200% of the federal poverty threshold group* (1). We also included child sex and adult‐to‐child ratio as control variables. Child sex reported by mothers was coded as *boy* (0) and *girl* (1). The adult‐child ratio was created based on the number of adults and children in the household at age 3, with higher values reflecting greater adult‐to‐child ratios. Mother's race/ethnicity was self‐reported and recoded into mutually exclusive categories by the FFCWS team. Mothers first reported their race (i.e., White, Black, Asian or Pacific Islander, American Indian, or Other) and then indicated whether they were of Hispanic/Latine origin. Individuals identified as Hispanic/Latine were given coding priority, resulting in four mutually exclusive categories: White, Non‐Hispanic; Black, Non‐Hispanic; Latine; and Others. These categories were then dummy‐coded for analysis purposes.

### 2.3 Analysis Plan

Preliminary analyses were conducted to examine descriptives, correlation, and missing data among the studied variables in R Version 4.1.1 (*nparcomp* package). Little's Missing Completely at Random (MCAR) test was not significant, suggesting that the data were missing completely at random (*χ* ^2^ (25,677) = 12,317, *p* > 0.05). We compared the analytic sample with complete data among demographic variables and found that non‐poor families were more likely to have missing data on household chaos at age 3 (*χ* ^2^ (1) = 6.82, *p* < 0.001) and age 5 (*χ* ^2^ (1) = 5.82, *p* = 0.02). Non‐poor families were also more likely to have missing data on parenting stress at age 3 (*χ* ^2^ (1) = 5.73, *p* = 0.02). Families with higher adult‐to‐child ratios were more likely to have missing data on household chaos at age 3 (*t* (3,901) = −4.36, *p* < 0.001) and behavioral self‐regulation at age 3 (*t* (2,780) = −2.12, *p* = 0.03). Families with lower adult‐to‐child ratios were more likely to have missing data on parenting stress at age 3 (*t* (85) = 3.88, *p* < 0.001). Missing data were addressed using Full Information Maximum Likelihood (FIML) estimation, which computed the likelihood for each individual based on all available information for that family (Enders 2022).

Previous studies examining household chaos, parenting stress, and behavioral self‐regulation have primarily relied on manifest variable approaches, such as creating composite sum or average scores. However, the manifest variable approach had several limitations given the strict assumptions, including tau‐equivalence, perfect measurement reliability (i.e., no estimated measurement error), and measurement invariance over time (Little 2024). These restrictions can either be relaxed or explicitly tested using latent variable models. Thus, we employed a latent variable approach, first establishing measurement invariance models separately for each construct across all three time points and then integrating them into a comprehensive model. Longitudinal measurement models in Mplus 8.7 were assessed across three time points for each variable, first separately and then together within the same model. Model fit was evaluated using Chi‐square and several fit indices, including Comparative Fit Index (CFI) > 0.90, Tucker‐Lewis Index (TLI) > 0.90, Root Mean Square Error of Approximation (RMSEA) < 0.08, and Standardized Root Mean Square Residual (SRMR) < 0.08, following standard cut‐offs recommended by Hu and Bentler (1999). After establishing a measurement model with good fit, we examined longitudinal measurement invariance across the three time points to provide evidence that these constructs were measured and interpreted consistently over time (Wu and Estabrook 2016). We began with a configural model using the Mplus default approach to scale the latent variables by fixing the first item loading to 1 and the latent variables' mean to 0 at the first time point. All thresholds were freely estimated, and residual variances were constrained to 1 in the configural model for identification. Given the latent response assumption of the threshold model, loadings are not comparable unless threshold equality constraints are also included. Therefore, we examined metric and scalar invariance in one model, which constrained both loadings and thresholds for all latent variables over time, and constrained the residual variances to 1 for items at age 3, and freely estimated residual variances for items at subsequent ages. Lastly, strict invariance was examined by constraining the residual variance to 1 across time for all items. To evaluate each test of measurement invariance, we used the Chi‐square difference test in combination with a change in CFI and RMSEA of less than 0.01.

After establishing longitudinal measurement invariance, we fit our hypothesized Cross‐Lagged Panel Model (CLPM) that included four control variables (i.e., family poverty status, child sex, adult‐child ratio, and maternal race). We first examined the CLPM model with all four covariates that were modeled as predictors of household chaos, parenting stress, and children's self‐regulation across all three time points. Non‐significant covariate paths were subsequently removed from the CLPM for parsimony purposes. To account for the ordinal nature of all indicators, we used the weighted least squares means and variance adjusted estimator (WLSMV) with theta parameterization to fit all latent variables. The WLSMV estimator used thresholds and latent response variables to account for the categorical nature of the item indicators.

## 3 Results

### 3.1 Measurement Models

Three models with nine latent variables were tested using the ordinal Confirmatory Factor Analysis to establish good fitting measurement models (Table 2). First, the five items measuring household chaos at three time points were modeled to establish the latent variables. The model fit the data well, *χ* ^2^ (72) = 386.17, *p* < 0.001, CFI = 0.97, TLI = 0.96, SRMR = 0.07, RMSEA = 0.03. Then, the four items representing parenting stress at three time points were modeled, yielding a good model fit, *χ* ^2^ (39) = 268.15, *p* < 0.001, CFI = 0.99, TLI = 0.98, SRMR = 0.02, RMSEA = 0.04. Lastly, 14 items assessing children's behavioral self‐regulation were modeled, and model fit was good, *χ* ^2^ (774) = 4086.49, *p* < 0.001, CFI = 0.94, TLI = 0.93, SRMR = 0.03, RMSEA = 0.05. Correlation among the nine latent variables can be found in Table 3.

**TABLE 2:** Fit indices for three longitudinal ordinal factor analysis models.

Models	Chi‐square (df)	CFI	TLI	SRMR	RMSEA	90% CI
1. Household Chaos	386.17*** (72)	0.97	0.96	0.07	0.03	[0.03, 0.04]
2. Maternal Parenting Stress	268.15*** (39)	0.99	0.98	0.02	0.04	[0.03, 0.04]
3. Behavioral Self‐regulation	4086.49*** (774)	0.94	0.93	0.03	0.05	[0.03, 0.032]

Abbreviations: CFI, Comparative Fit Index; RMSEA, Root Mean Squared Error of Approximation; SRMR, Standardized Root Mean Squared Residual; TLI, Tucker‐Lewis Index.

*p < 0.05, **p < 0.01, ***p < 0.001.

**TABLE 3:** Correlation table among studied latent variables (*N* = 4195).

		1	2	3	4	5	6	7	8	9
1	Behavioral Regulation (Y3)	1.00								
2	Behavioral Regulation (Y5)	0.66***	1.00							
3	Behavioral Regulation (Y9)	0.47***	0.59***	1.00						
4	Household Chaos (Y3)	−0.15***	−0.13***	−0.13***	1.00					
5	Household Chaos (Y5)	−0.20***	−0.20***	−0.18***	0.44***	1.00				
6	Household Chaos (Y9)	−0.11***	−0.13***	−0.14***	0.36***	0.39***	1.00			
7	Maternal Parenting Stress (Y3)	−0.34***	−0.31***	−0.21***	0.04	0.07*	0.05*	1.00		
8	Maternal Parenting Stress (Y5)	−0.32***	−0.44***	−0.27***	0.07^+^	0.14***	0.07*	0.71***	1.00	
9	Maternal Parenting Stress (Y9)	−0.26***	−0.28***	−0.33***	0.07^+^	0.12***	0.13***	0.51***	0.59***	1.00

*Note:* Y3, Focal child at age 3; Y5, Focal child at age 5; Y9, Focal child at age 9. ^+^ *p* < 0.01, *p < 0.05, **p < 0.01, ***p < 0.001.

### 3.2 Structural Model

**FIGURE 1:**
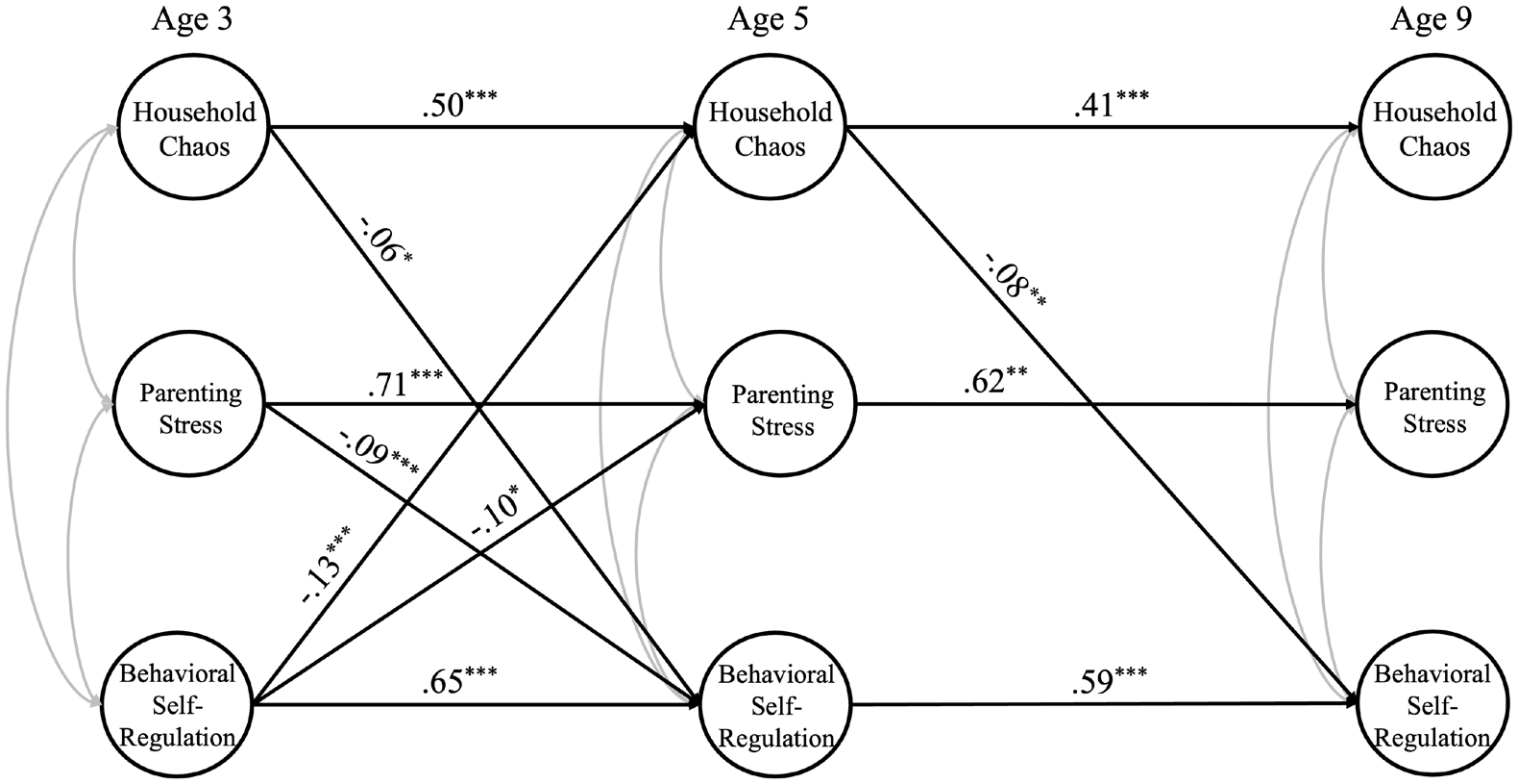
Cross‐lagged panel model with latent variables across three waves. *N* = 4195. *χ* ^2^ = 6773.83, df = 2581, CFI = 0.94, TLI = 0.94, RMSEA = 0.02, SRMR = 0.05. Only significant paths are reported and estimates shown are standardized coefficients. Controls included family poverty status, child sex, adult‐child ratio, and maternal race/ethnicity at age 3. *p < 0.05, **p < 0.01, ***p < 0.001.

After establishing partial measurement invariance for each latent variable (see Appendix S1), we fit the Cross‐Lagged Panel Model to examine the reciprocal relationships among household chaos, parenting stress, and children's behavioral self‐regulation from ages three to nine. The model presented good fit (CFI = 0.94, TLI = 0.94, RMSEA = 0.02, SRMR = 0.05; Figure 1, Table 4). All variables' auto‐regressive paths were significant over time. Results demonstrated significant bidirectional associations in early childhood, such that household chaos at age three predicted children's behavioral self‐regulation at age five (*β* = −0.06, *p* = 0.03), and children's behavioral self‐regulation at age three predicted household chaos at age five (*β* = −0.13, *p* < 0.001). Similarly, parenting stress at age three predicted children's behavioral self‐regulation at age five (*β* = −0.09, *p* < 0.001), and children's behavioral self‐regulation at age three predicted parenting stress at age five (*β* = −0.10, *p* < 0.001). We did not find significant bidirectional effects between household chaos and parenting stress. In middle childhood, household chaos at age five significantly predicted children's behavioral self‐regulation at age nine (*β* = −0.08, *p* = 0.003). However, no other significant associations were detected in middle childhood.

**TABLE 4:** Reciprocal relations among household chaos, maternal parenting stress, and behavioral self‐regulation.

	Structural Model *b* (SE)	95% CI
Auto regressive paths		
Household Chaos_age3_ → Household Chaos_age5_	0.50*** (0.04)	[0.32, 0.57]
Household Chaos_age5_ → Household Chaos_age9_	0.41*** (0.04)	[0.26, 0.46]
Maternal Parenting Stress_age3_ → Maternal Parenting Stress_age5_	0.71*** (0.02)	[0.66, 0.84]
Maternal Parenting Stress_age5_ → Maternal Parenting Stress_age9_	0.62*** (0.03)	[0.54, 0.72]
Behavioral Self‐regulation_age3_ → Behavioral Self‐regulation_age5_	0.65*** (0.02)	[0.49, 0.65]
Behavioral Self‐regulation_age5_ → Behavioral Self‐regulation_age9_	0.59*** (0.03)	[0.64, 0.88]
Lag effects		
Age 3 → Age 5		
Household Chaos_age3_ → Maternal Parenting Stress_age5_	0.05 (0.03)	[−0.01, 0.05]
Household Chaos_age3_ → Behavioral Self‐regulation_age5_	−0.06* (0.03)	[−0.04, −0.002]
Maternal Parenting Stress_age3_ → Household Chaos_age5_	0.02 (0.04)	[−0.10, 0.18]
Maternal Parenting Stress_age3_ → Behavioral Self‐regulation_age5_	−0.09*** (0.02)	[−0.11, −0.04]
Behavioral Self‐regulation_age3_ → Household Chaos_age5_	−0.13** (0.04)	[−0.47, −0.11]
Behavioral Self‐regulation_age3_ → Maternal Parenting Stress_age5_	−0.10*** (0.02)	[−0.17, −0.06]
Age 5 → Age 9		
Household Chaos_age5_ → Maternal Parenting Stress_age9_	0.04 (0.03)	[−0.01, 0.05]
Household Chaos_age5_ → Behavioral Self‐regulation_age9_	−0.08** (0.03)	[−0.07, −0.01]
Maternal Parenting Stress_age5_ → Household Chaos_age9_	−0.02 (0.03)	[−0.14, 0.08]
Maternal Parenting Stress_age5_ → Behavioral Self‐regulation_age9_	−0.005 (0.03)	[−0.06, 0.05]
Behavioral Self‐regulation_age5_ → Household Chaos_age9_	−0.05 (0.03)	[−0.25, 0.05]
Behavioral Self‐regulation_age5_ → Maternal Parenting Stress_age9_	−0.02 (0.03)	[−0.10, 0.04]
Residual (Co)variance		
Household Chaos_age3_ with Maternal Parenting Stress_age3_	−0.01 (0.03)	
Household Chaos_age3_ with Behavioral Self‐regulation_age3_	−0.09** (0.03)	
Maternal Parenting Stress_age3_ with Behavioral Self‐regulation_age3_	−0.33*** (0.02)	
Household Chaos_age5_ *with* Maternal Parenting Stress_age5_	0.07 (0.05)	
Household Chaos_age5_ *with* Behavioral Self‐regulation_age5_	−0.06 (0.05)	
Maternal Parenting Stress_age5_ *with* Behavioral Self‐regulation_age5_	−0.30*** (0.04)	
Household Chaos_age9_ *with* Maternal Parenting Stress_age9_	−0.09* (0.04)	
Household Chaos_age9_ *with* Behavioral Self‐regulation_age9_	−0.03 (0.03)	
Maternal Parenting Stress_age9_ *with* Behavioral Self‐regulation_age9_	−0.22*** (0.03)	
*R* ^2^		
Household Chaosa_age3_		0.19
Household Chaosa_age5_		0.30
Household Chaosa_age9_		0.22
Maternal Parenting Stress_age3_		0.01
Maternal Parenting Stress_age5_		0.56
Maternal Parenting Stress_age9_		0.41
Behavioral Self‐regulation_age3_		0.05
Behavioral Self‐regulation_age5_		0.49
Behavioral Self‐regulation_age9_		0.40
Fit statistics		
*χ* ^2^	6721.64	
df	2581	
CFI	0.94	
TLI	0.94	
RMSEA	0.02	
SRMR	0.05	

*Note:* Estimates shown are standardized coefficients. Confidence intervals represent 95% CI. Controls include family poverty status, child sex, adult‐child ratio, and maternal race at age 3. Subscripts indicate the time of measurement across three time points: age 3, age 5, and age 9. *R* ^2^ values indicate the proportion of variance explained in each latent construct.

Abbreviations: CFI, Comparative Fit Index; df, degree of freedom; RMSEA, Root Mean Squared Error of Approximation; SRMR, Standardized Root Mean Squared Residual; TLI, Tucker‐Lewis Index.

*p < 0.05, **p < 0.01, ***p < 0.001.

Regarding significant pathways for covariates, a higher adult‐child ratio at age 3 was associated with lower household chaos at age 3 (*β* = −0.10, *p* = 0.001). Children from families at or above 200% of the federal poverty threshold group at age 3 experienced lower levels of household chaos at age 3 (*β* = −0.35, *p* < 0.001), and at age 9 (β = −0.14, *p* < 0.001), lower levels of parenting stress at age 3 (*β* = −0.09, *p* < 0.001), and higher levels of behavioral self‐regulation at age 3 (*β* = 0.21, *p* < 0.001), compared to children from families below 200% of the federal poverty threshold group. Girls demonstrated higher behavioral self‐regulation at age 3 (*β* = 0.09, *p* < 0.001), age 5 (*β* = 0.05, *p* = 0.004), and age 9 (*β* = 0.08, *p* < 0.001) than boys. Children of Black mothers experienced higher household chaos at age 3 (*β* = 0.14, *p* < 0.001) and higher behavioral self‐regulation at age 9 (*β* = 0.08, *p* = 0.003), compared to children of White mothers. Children of Latine mothers experienced lower parenting stress at age 9 (*β* = −0.10, *p* < 0.001), and exhibited higher behavioral self‐regulation at age 9 (*β* = 0.10, *p* < 0.001), compared to children of White mothers.

## 4 Discussion

This study aims to elucidate the interactions among household chaos, parenting stress, and children's behavioral self‐regulation, providing empirical evidence for the transactional models and theoretical frameworks (Sameroff 2009; Wachs and Evans 2010). Our results identified early bidirectional effects between household chaos and behavioral self‐regulation, along with a reciprocal association between parenting stress and behavioral self‐regulation. While these results were exclusive to early childhood, unidirectional associations between household chaos and behavioral self‐regulation were found between early and middle childhood.

### 4.1 Household Chaos and Children's Behavioral Self‐Regulation

The bidirectional effect between household chaos and children's behavioral self‐regulation during early childhood aligned partially with our first hypothesis. This pattern is consistent with prior literature demonstrating that household chaos can disrupt children's development of behavioral self‐regulation by undermining their ability to establish stable routines and develop effective behavioral coping strategies (Coldwell et al. 2006; Liu, Zhang et al. 2024; Selman and Dilworth‐Bart 2023). Furthermore, this finding corroborates the relational development systems metatheory, which posits that children's development is shaped by interactions between the child and their environment (Lerner et al. 2006). Behavioral self‐regulation develops rapidly in early childhood and is particularly sensitive to environmental contexts, especially the level of structure and predictability within the home (McClelland et al. 2015). Household chaos introduces high unpredictability, which could disrupt children's ability to engage in higher‐order cognitive processes necessary for managing behaviors (Andrews, Atkinson, et al. 2021; Andrews, Dunn, et al. 2021; Vernon‐Feagans et al. 2016). This disruption could hinder the internalization of self‐regulatory skills, leaving children more vulnerable to behavioral challenges.

Conversely, children's early behavioral self‐regulation at age three also predicted lower levels of household chaos at age five, which provides empirical evidence to support the transactional framework (Sameroff 2009). This highlights that children are active agents within the home environment. Children are not passive recipients of their contexts, but their self‐regulatory behaviors could actively shape and influence the family dynamics. Specifically, children who exhibit higher levels of behavioral self‐regulation may help reduce household chaos by adhering to household routines, while those who struggle with behavioral self‐regulation may be more likely to engage in disruptive behaviors, such as tantrums, non‐compliance, or refusing to follow external expectations, which could exacerbate household chaos (Crespo et al. 2019; Jaffee et al. 2012).

As children transition into middle childhood, our findings revealed a salient and unidirectional association, such that household chaos at age five predicted behavioral self‐regulation at age nine. This finding aligns with prior research indicating that chronic exposure to chaotic environments could create long‐term negative stressors, impeding children's ability to develop critical self‐regulatory skills across developmental stages (Evans and Kim 2013). Prolonged exposure to noise, disorganization, and unpredictability could overwhelm children's ability to develop coping strategies, leaving them less equipped to manage their behaviors effectively as they age (Andrews, Atkinson, et al. 2021; Andrews, Dunn, et al. 2021). Surprisingly, contrary to our hypothesis, we did not find a reciprocal association during the transition from early to middle childhood. The absence of a predictive relationship between behavioral self‐regulation at age five and household chaos at age nine could be attributed to several factors. As children grow older, their direct influence on family dynamics may diminish as their behaviors become less central to the household functioning. During middle childhood, increased autonomy and exposure to external environments, such as school context and peer interactions, play a prominent role in shaping self‐regulation (Chahl et al. 2024; Morín and Keulers 2024). Children's behavioral self‐regulation tends to become more prominent in external contexts rather than at home (Sameroff 2010), which may reduce the likelihood that their behaviors contribute to household chaos.

Additionally, household chaos tends to be relatively stable over time, often driven by persistent structural factors such as poverty, which increases the likelihood of experiencing instability through limited resources, overcrowded living conditions, and unpredictable routines (Wachs and Evans 2010). These systematic challenges may not be easily mitigated by improvements in a child's behavior, as the broader structural stressors continue to influence overall family dynamics. Importantly, the experience and interpretation of household chaos are not culturally universal, such that what is perceived as “chaos” in one context may reflect adaptive flexibility or culturally normative practices in another, particularly among families navigating economic hardship or living in multigenerational households (Cherry and Gerstein 2023; Wachs and Corapci 2003). Our racially and socioeconomically diverse sample offers valuable insight into how household chaos could emerge and function across different sociocultural and economic contexts. These findings highlight the importance of considering household chaos as a dynamic, contextually embedded construct shaped by families' social location and lived realities.

### 4.2 Parenting Stress and Children's Behavioral Self‐Regulation

Our findings revealed a bidirectional association between parenting stress and children's behavioral self‐regulation in early childhood, partially aligned with our second hypothesis. These results highlight the mutual influence within the family system, consistent with the transactional framework, which emphasizes dynamic, reciprocal interactions that contribute to cyclical development patterns (Sameroff 2009). Specifically, our findings suggest that heightened parenting stress may introduce stressors during parent–child interactions in early childhood, which is a critical developmental period when children's higher‐order cognitive regulation is highly sensitive and malleable (Blair 2010). The inconsistency and frustration stemming from these interactions could hinder children's ability to manage behaviors effectively and develop dysregulated behaviors (Cook et al. 2024; Gee et al. 2018; Suh and Luthar 2020), as they may receive negative cues or experience parental frustration during these interactions.

Notably, children's better behavioral self‐regulation further reduced parenting stress, highlighting the positive feedback loop within family dynamics. When children manage their behaviors effectively, it tends to lessen the demands on parents, leading to smoother interactions and fostering more positive synchrony in parent–child behaviors (Davis et al. 2017). As a result, the parenting process becomes less stressful, reinforcing a cycle of more positive and cooperative interactions. In contrast, dysregulated children may provoke more challenging behavioral problems (Perry et al. 2018). These heightened demands increase parental stress and create a vicious cycle of heightened tension and negative interactions through behavioral difficulties (Deater‐Deckard 1998).

However, the bidirectional relationship between parenting stress and children's behavioral self‐regulation was not observed in middle childhood. This finding should be interpreted with caution. One possible explanation is that this may be an artifact of the data, as measures of parenting stress and behavioral self‐regulation over a four‐year period may not capture the moment‐to‐moment interactions where stress and dysregulation manifest most acutely (Li et al. 2024). Parenting stress in response to dysregulated behaviors likely emerges in specific, immediate interactions rather than being sustained over such a long period. Moreover, as children transition into middle childhood, they spend increasing time in external contexts, such as school, extracurricular activities, and peer socialization, where their social goals and behaviors are shaped by interactions outside the home (Rodkin et al. 2013). This developmental shift may reduce their exposure to the home environment and thus lessen the direct impact of parenting stress on their self‐regulation. Notably, our control variables suggested strength‐based patterns, such that children of Latine mothers exhibited lower parenting stress and higher behavioral self‐regulation at age nine compared to those of White mothers. This suggests the importance of contextualizing parenting stress processes within culturally grounded strengths and structural inequities (García‐Coll et al. 1996), particularly given the structural challenges and systematic barriers that minoritized families could face (Garland McKinney and Meinersmann 2023).

### 4.3 Household Chaos and Parenting Stress

Contrary to our third hypothesis, bidirectional effects between household chaos and parenting stress were not observed in either early or middle childhood. Several plausible explanations may account for these null findings. First, the six‐year interval between measurement points may have obscured more dynamic, short‐term interactions and other contextual factors during this period, such as interparental conflict (Camisasca et al. 2016) and violence (Zhang et al. 2024), which could also influence fluctuations in parenting stress. Second, the measure of household chaos primarily reflects structural instability, such as clutter, crowd, and noise, which might not uniformly affect parenting stress across all families. As supported by our covariate results, household chaos was more prevalent in families experiencing poverty and in households of Black mothers. These disparities highlight that home environment and parenting stress could be embedded within broader socioeconomic and structural barriers that could limit access to stability and predictability at home (Evans et al. 2005; Garland McKinney and Meinersmann 2023). It is also possible that over this time, parents developed adaptive coping strategies to manage chronic environmental chaos (Skinner and Zimmer‐Gembeck 2016). Lastly, prior research has found an association between household chaos and general psychological distress (Bodrij et al. 2021), raising the possibility that chaotic environments could impact overall stress above and beyond parenting stress. It is possible that parenting stress may only represent part of the broader psychological distress that parents have been experiencing (Pinto et al. 2019).

### 4.4 Limitations and Future Directions

One limitation of this study arises from the scale used in the secondary data, which, with its limited items, may not adequately capture the multifaceted nature of household chaos. Future research should consider employing a multi‐informant and multi‐method strategy to more effectively address the diverse dimensions of chaos. This approach would facilitate the incorporation of various perspectives (e.g., from parents and children) and methodologies (e.g., observational data, parent self‐reports). Further, the current study was unable to use the full scale of the Parenting Stress Inventory due to limited items available in the FFCWS dataset. Future studies should adopt a full scale of PSI to capture empirically derived domains, including Parental Distress, Parent–Child Dysfunctional Interaction, and Difficult Child (Haskett et al. 2006; Whiteside‐Mansell et al. 2007). Although the current study includes a racially and socioeconomically diverse cohort of families primarily from urban settings in the early 2000s, its demographic and geographic limitations constrain the generalizability of findings to contemporary, nationally representative populations. Future research should draw on more diverse and contemporary samples that reflect a broader array of family structures, cultural backgrounds, and living conditions. Moreover, future work should incorporate culturally responsive approaches to better capture how systemic and contextual factors shape the development of household chaos, parenting stress, and children's behavioral self‐regulation across racially and ethnically diverse families. Such work could help disentangle how these processes function similarly or differently across groups, advancing both theoretical precision and equity in developmental science.

Additionally, considering the significant changes in digital media consumption over the past two decades, it is crucial to include variables such as screen time in future datasets. Multiple devices playing simultaneously can significantly increase the noise level in a home, contributing to the overall sense of disorganization and disruption. As family and children's screen time has increased dramatically, understanding its role in the context of understanding household chaos and child self‐regulation becomes essential. Recent data that account for these modern variables will help ensure that the findings are relevant to today's socio‐technological environment and more accurately reflect the current challenges and realities families face. Furthermore, this study used the Cross‐Lagged Panel Model, which did not distinguish between‐person and within‐person variance, potentially biasing the interpretations of the results (Hamaker et al. 2015). Future studies should consider using the Random‐Intercept Cross‐Lagged Panel Model (Berry and Willoughby 2017; Lucas 2023), which accounts for stable inter‐individual differences and more accurately captures within‐person fluctuations over time. Lastly, while the current study focused on the bidirectional associations among household chaos, parenting stress, and children's behavioral self‐regulation, prior research suggests that other parenting relational processes, such as parental acceptance, sensitivity, and responsivity, may serve as underlying processes linking early contextual chaos and children's self‐regulation (Andeweg et al. 2024; Coldwell et al. 2006; Vernon‐Feagans et al. 2016). Future research should explore these additional relational processes to further elucidate the underlying pathways of chaos and behavioral self‐regulation while accounting for their bidirectional nature.

### 4.5 Implications

The findings from this study align closely with and expand upon the foundational ideas presented in Evans and Wachs' (2010) work, which posits how context and children's behaviors are mutually influenced. By examining household chaos, parenting stress, and children's behavioral self‐regulation, this study provides robust empirical support for these theoretical assertions. It highlights how chaotic environments are linked with children's ability to regulate themselves. Our findings suggest a dynamic and ongoing exchange between household chaos and parenting stress, where each component simultaneously shapes and is shaped by the other. Such findings underscore the complexity of environmental impacts on child development. In addition, the study's findings enrich the transactional model proposed by Sameroff (2009), which emphasizes the mutual influences of contextual and relational processes on child development. By utilizing longitudinal data to illustrate these interactions over the transition from early to middle childhood, the research provides evidence of how stability and change in child behavioral regulation are influenced by an evolving home environment and parenting stress. This approach encourages future research to consider child effects within the broader spectrum of environmental and relational dynamics, potentially leading to more effective strategies for fostering positive developmental outcomes in children.

Intervention programs aimed at alleviating household chaos may be essential in fostering child development, particularly during the critical early childhood years. These programs not only modify the environmental factors contributing to chaos but also equip families with strategies and resources to enhance functioning and reduce parenting stress. Tailored interventions are needed to address the unique needs of each family and incorporate elements like home organization strategies, time management skills, and stress reduction techniques. For example, the Structuring the Home to Induce a Nurturing Environment (SHINE) intervention exemplifies a structured approach with a series of four home visits, each spaced 2 weeks apart, targeting different aspects of household management (Andeweg et al. 2022). The themes of these visits are strategically designed to tackle key elements of household chaos: establishing Family Routines and Weekly Schedules, Organizing Materials in the Home, and Reducing Noise.

To alleviate parenting stress, the Mayo Clinic Family Stress Resource Center's “Family Stress Relief Initiative” focuses on mindfulness and stress management techniques. This program includes sessions on relaxation, problem‐solving, and establishing a calm and nurturing home environment (Mayo Foundation for Medical Education and Research 2020). Such prevention programs could be extended into community and educational settings, enhancing their impact. For example, schools can help bring resources and training opportunities to parents on maintaining routines and discipline that mirror the classroom setting, further reducing chaos at home and parenting stress. For working on children's behavioral regulation, incorporating simple activities, such as games that require turn‐taking, following rules, or managing frustration, would be beneficial at home. Activities could also include mindfulness exercises such as guided breathing or yoga (Razza et al. 2015). These activities not only make the learning process fun but also provide practical tools for children to manage their impulses and emotions.

## 5 Conclusion

This study is among the first to examine a comprehensive transactional model involving household chaos, parenting stress, and children's behavioral self‐regulation. The findings emphasize the importance of reducing household chaos and improving parental stress management strategies during early childhood. By highlighting the interconnectedness within family dynamics, these findings suggest interventions should be designed to address specific challenges faced by families experiencing high levels of chaos and parenting stress to promote overall family functioning holistically.

## Conflicts of Interest

The authors declare no conflicts of interest.

## Supporting information


Appendix S1.

## Data Availability

The scripts to reproduce all study findings are available upon request. The current manuscript uses the publicly available data from the Future of Families and Child Wellbeing Study, which can be downloaded at https://ffcws.princeton.edu/documentation.
