# Supplementary material for: Reciprocal Relationships Among Household Chaos, Parenting Stress, and Children's Behavioral Self‐Regulation From Early to Middle Childhood
Source: Fam Process. 2025 Jun 17;64(2):e70053. doi: 10.1111/famp.70053 (PMC12174505; doi:10.1111/famp.70053)
Supplement: Supplementary file 1 — Appendix S1. [file FAMP-64-0-s001.docx]

**Supplemental Material**

**Longitudinal Measurement Invariance**

After establishing a good fitting measurement model, we further examined longitudinal measurement invariance across three time points together in a single model (STable 1). The configural invariance model had a good fit, χ^2^ (2,172) = 5882.52, *p* < .001, CFI = .95, TLI = .95, SRMR = .05, RMSEA = .02, and was used as the baseline model for subsequent measurement invariance tests. We then examined metric and scalar invariance together in one model. The metric and scalar invariance model had an acceptable fit, χ^2^ (2,256) = 10,391, *p* < .001, CFI = .89, TLI = .89, SRMR = .05, RMSEA = .03. Because the change in CFI was greater than .001 between the configural invariance model and the metric and scalar invariance model and the chi-square difference test was statistically significant between the configural and metric/scalar invariance models, χ^2^(84) = 4508.5, *p* < .05, full metric and scalar invariance was not supported. Based on modification indices, we released the constraints of 7 thresholds, allowing them to be freely estimated in a partial metric and scalar model. ​​Because the partial metric and scalar model was not nested within the configural model, a chi-square difference test was not conducted. The partial metric and scalar model yielded good model fit, χ^2^ (2,021) = 6241.34, *p* < .001, CFI = .94, TLI = .93, SRMR = .05, RMSEA = .02. The change in CFI and RMSEA were both less than 0.01 (ΔCFI = 0.01, ΔRMSEA = 0.01); thus, longitudinal partial metric and scalar invariance were established.

Lastly, the strict invariance model showed acceptable model fit, χ^2^ (2,288) = 9431.40, *p* < .001, CFI = .91, TLI = .91, SRMR = .05, RMSEA = .03. However, the comparison between the partial metric and scalar model and the strict model did not pass the criteria that the change in CFI was larger than 0.01(ΔCFI = 0.03) and the chi-square difference test was statistically significant between the configural and metric/scalar invariance models, χ^2^(267) = 3190.1, *p* < .05; strict invariance was not supported. Based on the model modification indices, we released the constraints of 9 residuals and allowed them to be freely estimated in the partial strict model. ​​Because the partial strict model was not nested within the partial metric and scalar model, a chi-square difference test was not conducted. The partial strict model yielded a good model fit, χ^2^ (2,279) = 8041.65, *p* < .001, CFI = .93, TLI = .92, SRMR = .05, RMSEA = .02. The change in CFI and RMSEA were both less than 0.01 (ΔCFI = 0.01, ΔRMSEA = 0.00); thus, longitudinal partial strict invariance was established.

**Table S1**

*Fit Indices for Longitudinal Measurement Invariance*

| Models | χ^2^ (*df*) | CFI | ΔCFI | TLI | RMSEA | ΔRMSEA | SRMR |
| --- | --- | --- | --- | --- | --- | --- | --- |
| Configural Model | 5882.52^***^ (2,172) | .95 | — | .95 | .02 | — | .05 |
| Metric & Scalar Model | 10,391^***^ (2,256) | .89 | .06 | .89 | .03 | .01 | .05 |
| Partial Metric & Scalar Model | 6241.34^***^ (2,021) | .94 | .01 | .93 | .02 | .00 | .05 |
| Strict Model | 9431.40^***^ (2,288) | .91 | .03 | .91 | .03 | .01 | .05 |
| Partial Strict Model | 8041.65^***^ (2,279) | .93 | .01 | .92 | .02 | .00 | .05 |

*Note.* CFI = Comparative Fit Index; TLI = Tucker-Lewis Index; RMSEA = Root Mean Squared Error of Approximation; SRMR = Standardized Root Mean Squared Residual.

Partial Metric and Scalar Model released constrained of 7 thresholds. Partial Strict Model released constrained of 9 residual variances.

^*^*p* < .05, ^**^*p* < .01, ^***^*p* < .001.

**Table S2**

*Household Chaos Confirmatory Factor Analysis Loadings at Ages 3, 5, and 9*

| Household Chaos Items | Longitudinal CFA Factor Loading | | |
| --- | --- | --- | --- |
|  | Year 3 | Year 5 | Year 9 |
| 1. Is the inside of the home crowded? | .86 | .84 | .80 |
| 1. Are all visible rooms of house/apartment noticeably cluttered? | .90 | .93 | .86 |
| 1. Are all visible rooms of the house/apartment dirty or not reasonably cleaned? | .82 | .85 | .87 |
| 1. Is the environment inside the home unsafe for young children? | .57 | .61 | .61 |
| 1. Is the house or apartment overly noisy? | .67 | .67 | .58 |

*Note*. CFA = Confirmatory Factor Analysis. Standardized loadings were reported.

**Table S3**

*Maternal Parenting Stress Confirmatory Factor Analysis Loadings at Ages 3, 5, and 9*

| Household Chaos 6 Items | Longitudinal CFA Factor Loading | | |
| --- | --- | --- | --- |
|  | Year 3 | Year 5 | Year 9 |
| 1. Being a parent is harder than I thought it would be. | .60 | .62 | .63 |
| 1. I feel trapped by my responsibilities as a parent. | .68 | .70 | .70 |
| 1. I find that taking care of my child(ren) is much more work than pleasure. | .65 | .66 | .73 |
| 1. I often feel tired, worn out, or exhausted from raising a family. | .64 | .65 | .67 |

*Note*. CFA = Confirmatory Factor Analysis. Standardized loadings were reported.

**Table S4**

*Behavioral Self-regulation Confirmatory Factor Analysis Loadings at Ages 3, 5, and 9*

| Behavioral Self-Regulation Items | Longitudinal CFA Factor Loading | | |
| --- | --- | --- | --- |
|  | Year 3 | Year 5 | Year 9 |
| 1. Child cries a lot. | .57 | .52 | .61 |
| 1. Child acts too young for his/her age. | .41 | .51 | .53 |
| 1. Can’t concentrate, can’t pay attention for long. | .61 | .64 | .73 |
| 1. Can’t sit still, is restless or hyperactive. | .70 | .70 | .75 |
| 1. Clings to adults or is too dependent. | .42 | .42 | .49 |
| 1. Destroys his/her own things. | .65 | .59 | .76 |
| 1. Disobedient at home. | .65 | .60 | .67 |
| 1. Gets in many fights. | .62 | .62 | .71 |
| 1. Physically attacks people. | .65 | .67 | .75 |
| 1. Screams a lot. | .67 | .62 | .74 |
| 1. Has temper tantrums or hot temper. | .72 | .68 | .80 |
| 1. Child wants a lot of attention | .50 | .55 | .71 |
| 1. Child does not feel guilty after misbehaving. | .46 | .44 | .60 |
| 1. Is stubborn, sullen, or irritable. | .62 | .66 | .71 |

*Note*. CFA = Confirmatory Factor Analysis. Standardized loadings were reported.
